# P030 Inflammatory or atherosclerotic? A diagnostic journey from acute coronary syndrome to large vessel vasculitis

**DOI:** 10.1093/rap/rkag113.060

**Published:** 2026-11-03

**Authors:** James Cai, Spencer Ellis

**Affiliations:** University College Hospital London, London, United Kingdom; Lister Hospital, Stevenage, United Kingdom

## Abstract

**Introduction:**

Takayasu arteritis is a large-vessel vasculitis (LVV) affecting the aorta and its major branches. Coronary artery involvement is uncommon but can cause substantial cardiovascular morbidity. Recognition is particularly challenging when patients present with features more typical of atherosclerotic coronary artery disease. We report a case of Takayasu arteritis presenting with critical coronary ostial stenosis requiring urgent coronary artery bypass grafting. The case highlights the diagnostic challenge of distinguishing inflammatory vasculopathy from atherosclerotic disease, the value of bedside examination alongside multimodal imaging and histopathology in establishing the diagnosis, and the importance of timely immunosuppression to optimise long-term vascular outcomes.

**Case description:**

A previously fit-and-well White British 46-year-old woman presented to the Chest Pain Clinic in May 2013 with right-sided chest pain radiating to the right arm after approximately 10 minutes of exercise. Symptoms were reproducible on exertion, relieved by rest, and progressed to angina on hills and during emotional stress. Her only significant cardiovascular risk factor was a previous smoking history (15 pack-years, ceased 9 years prior); she had no diabetes, hypertension or hypercholesterolaemia.

ECG demonstrated sinus rhythm with ST changes in V4-6 and non-specific changes in leads II, III, aVF. Coronary angiogram revealed critical stenosis at the ostium of the left mainstem coronary artery, with minor disease at the LAD origin and right coronary ostium, prompting urgent coronary artery bypass grafting in June 2013.

During surgery, inflammation of the aorta and coronary adventitia with adhesions was noted, raising concern for a vasculitic process. Histopathology later confirmed active aortitis. Laboratory investigations showed elevated inflammatory markers (CRP 36 mg/L, ESR 108mm/hr). Autoantibody testing demonstrated a weakly positive speckled ANA, with negative ENA, dsDNA, rheumatoid factor, ANCA and syphilis screen.

On rheumatology review in October 2013, examination revealed a blood pressure discrepancy (150/42 mmHg left arm vs 184/40 mmHg right arm), a reduced left radial pulse, an abdominal aortic bruit and a wide pulse pressure suggestive of aortic regurgitation. PET-CT demonstrated inflammatory activity extending from the aortic root to the diaphragmatic hiatus. A diagnosis of Takayasu’s arteritis was made and treatment commenced with prednisolone 40mg daily and methotrexate 15mg weekly.

She subsequently experienced several disease flares heralded by right-sided chest pain, requiring escalation to mycophenolate mofetil and subsequently tocilizumab. Her disease is currently well controlled, with ongoing surveillance for asymptomatic moderate-to-severe aortic regurgitation.

**Discussion:**

Coronary artery involvement is an uncommon but potentially life-threatening manifestation of Takayasu arteritis, occurring in approximately 9–11% of cases. When present, disease most commonly affects the coronary ostia, reflecting extension of aortic inflammation into the proximal coronary arteries. This pattern differs from conventional atherosclerotic coronary artery disease and may be overlooked when patients present with typical symptoms of myocardial ischaemia.

Our case highlights the diagnostic challenges posed by Takayasu arteritis presenting primarily with coronary disease and the importance of bedside assessment alongside laboratory and imaging investigations. While autoimmune serology was largely non-contributory, a subsequent careful vascular examination identified pulse asymmetry, inter-arm blood pressure discrepancy, an abdominal bruit and evidence of aortic regurgitation, substantially increasing clinical suspicion for large vessel vasculitis. These findings prompted PET-CT imaging, which confirmed active aortic inflammation and defined disease extent. Coronary angiography, histopathology and serial cardiovascular imaging each contributed distinct information that influenced diagnosis, treatment and long-term monitoring. Focused clinical examination was the key to maintaining momentum in establishing the diagnosis.

Although surgery preceded the diagnosis of LVV it is important to note that immunosuppression alone may be insufficient in patients with advanced coronary involvement, and surgical or percutaneous revascularisation may be required alongside disease-directed therapy. The optimal timing of intervention relative to disease activity remains uncertain.

Questions for discussion include whether earlier recognition of inflammatory aortitis could have altered the patient’s clinical course, how clinicians can better distinguish inflammatory coronary ostial disease from atherosclerotic disease at presentation, and how best to integrate investigations with clinical assessment when monitoring disease activity to guide treatment escalation in Takayasu arteritis.

**Key learning points:**

1. Critical coronary ostial stenosis may be the presenting manifestation of Takayasu arteritis.

2. Severe coronary disease in younger patients with limited cardiovascular risk factors should prompt consideration of inflammatory aortitis and large vessel vasculitis.

3. Pulse asymmetry, blood pressure discrepancies and persistently elevated inflammatory markers may provide important diagnostic clues.

4. PET-CT complements conventional vascular imaging by defining disease extent and inflammatory activity.

5. Once fixed fibrotic stenosis is present, revascularisation is required, typically with coronary artery bypass grafting.

6. Revascularisation may be unsuccessful, particularly when inflammatory disease is not under satisfactory control.

7. Coronary artery and aortic valve involvement are important causes of long-term morbidity in Takayasu arteritis.

